# The Notification of Tuberculosis

**Published:** 1901-08-10

**Authors:** 


### The Hospital

A JOURNAL OF

The Medical Sciences and Hospital Administration.

VOL. XXX.—NO. 776.] EDITED BY SIR HENRY BURDETT, K.C.B., AND SOLOMON C. SMITH, M.D., M.R.C.P. [AUGUST 10, 1901.

### THE NOTIFICATION OF TUBERCULOSIS.

THE most important of the practical questions brought before the public mind by the proceedings of the Congress on Tuberculosis is that of the advantages which might be gained by the "notification" of the disease, when these are duly weighed against the difficulties which might interfere with the efficient carrying out of any such procedure, and against the hardships which it might sometimes bring in its train. The late Sir Richard Thorne Thorne, whose official position had required him carefully to consider the subject, discussed it at some length in the Harben Lectures which he delivered in 1898, and arrived at conclusions altogether adverse to the proposal; but we are now told, by Sir William Broadbent and others, that Sir Richard Thorne's objections were based upon insufficient and (shocking to relate) upon *a priori* grounds, and that they must be entirely disregarded by persons of true enlightenment.

We are assured that as soon as a patient was notified as being the subject of tuberculosis, especially, we suppose, of pulmonary tuberculosis, he or she would become an object of solicitude, not only to medical officers of health and such like people, but also to associations of benevolent individuals, male and female, and would be visited from time to time by volunteer instructors in the art of avoiding the diffusion of contagion. This art, as Sir William Broadbent has himself declared, "practically resolves itself into the destruction of the sputa," and it is abundantly plain that the degree in which this end could be secured by the aid of notification would be the measure of the real utility of the notification itself.

In the case of a patient in humble life, who is either the inmate of a hospital or confined to bed at home, or even to the house, and who, in the last-mentioned cases, is under medical care, it may be assumed that the destruction of the sputa is even now attended to in the great majority of instances. It would certainly always be enjoined by the doctor, and would probably not be neglected by the relatives, if they were once made to understand its importance as a measure for their own security. There would be little practical difficulty, as a general rule, in bringing the condition of any such sufferer under the notice of any benevolent organisation which the locality might afford, and in this way the services of visitors might be obtained, and the advice already given by the doctor duly supplemented and enforced by their friendly counsels. But, in actual life, it is probable that the chief danger to the public arises during the ambulatory stages of the disease, when the patient is still following an occupation, or even before his cough or other symptoms have become sufficiently serious to induce him to take medical advice at all. When such advice is taken, how often, in the actual circumstances of the medical attendance which is provided for the working classes by the intelligence of their "leaders," will the club doctor or the "dispensary" doctor possess the leisure, the apparatus, or even the skill and knowledge which are required for the formation of a trustworthy conclusion upon the basis of a microscopic examination of the sputa? In former times there would have been an objection to the notification of the early stages of phthisis which would have been justly felt to be insuperable, arising from the fact that the disease was then regarded, even in its early stages, as hopelessly incurable, and that it was the constant endeavour of practitioners to avoid, as far as possible, the utterance of a name which the patients and their friends would alike have regarded as a death warrant. This objection no longer holds good, although, even now, it is highly probable that the chances of cure are much over-estimated by the public, and that they practically exist only for persons whose circumstances admit of a systematic regulation of the future life only attainable by patients of independent means, and which is only attained, as a matter of fact, by those who, in addition to the means, possess sufficient resolution to employ them for the purpose. Sir Richard Thorne pointed out that, although many urban and other authorities had sought the sanction of the Local Government Board to the introduction into local Acts of clauses authorising the notification of phthisis, not one of them had ever sent a satisfactory reply to the inquiry how they proposed to secure the notification, and how they proposed to employ, when they had obtained them, the facts which notification would reveal. It cannot be doubted that the notification of all, or of the great majority of, the occurring cases of phthisis, especially in its early stages, and still more especially if followed by the observance of proper precautions, would tend to bring about the extinction of the disease. In this view of the case, as a pious opinion, we are content to share. Nevertheless, we live in a complicated world, governed by many and oftentimes by apparently opposing forces; and, before we attempt to carry the pious opinion into practice, we should like to consider carefully the provisions of the Act of Parliament which would be required for the purpose, and the nature of the inspection and of the sanctions by which alone these provisions could be enforced.

